# The Impact of Hedgehog Signaling Pathway on DNA Repair Mechanisms in Human Cancer

**DOI:** 10.3390/cancers7030839

**Published:** 2015-07-21

**Authors:** Erhong Meng, Ann Hanna, Rajeev S. Samant, Lalita A. Shevde

**Affiliations:** Department of Pathology, Comprehensive Cancer Center, University of Alabama at Birmingham, WTI320D, 1824 6th Avenue South, Birmingham, AL 35233, USA; E-Mails: mengerhong@hotmail.com (E.M.); annhanna@uab.edu (A.H.); rsamant@uab.edu (R.S.S.)

**Keywords:** Hedgehog, cancer, DNA repair, drug resistance

## Abstract

Defined cellular mechanisms have evolved that recognize and repair DNA to protect the integrity of its structure and sequence when encountering assaults from endogenous and exogenous sources. There are five major DNA repair pathways: mismatch repair, nucleotide excision repair, direct repair, base excision repair and DNA double strand break repair (including non-homologous end joining and homologous recombination repair). Aberrant activation of the Hedgehog (Hh) signaling pathway is a feature of many cancer types. The Hh pathway has been documented to be indispensable for epithelial-mesenchymal transition, invasion and metastasis, cancer stemness, and chemoresistance. The functional transcription activators of the Hh pathway include the GLI proteins. Inhibition of the activity of GLI can interfere with almost all DNA repair types in human cancer, indicating that Hh/GLI functions may play an important role in enabling tumor cells to survive lethal types of DNA damage induced by chemotherapy and radiotherapy. Thus, Hh signaling presents an important therapeutic target to overcome DNA repair-enabled multi-drug resistance and consequently increase chemotherapeutic response in the treatment of cancer.

## 1. Introduction

DNA encounters insults on its native structure and sequence throughout the life span of a cell. There are multiple DNA repair pathways in cells; these maintain the integrity and offer protection from an extremely large variety of endogenous and exogenous agents [1,2,3]. Given the paradoxical need for the accumulation of such mutations for carcinogenesis and tumor cell survival, cancer cells have evolved methods to selectively allow for unchecked proliferation and resistance to DNA damaging agents. Cancer cells foster mutations in themselves to override cellular regulation of pathways responsible for restriction of growth, survival, and induction of therapy-resistance. In addition to cancer-driven mutations, continuous errors occur during the process of DNA replication. Small-scale errors such as single base changes are largely uninfluential to the integrity of DNA; however, major un-repaired changes like double stranded breaks can cause catastrophic outcomes. Aberrations due to endogenous and exogenous sources on the DNA are mainly corrected through five major repair pathways: mismatch repair (MMR), nucleotide excision repair (NER), direct repair, base excision repair (BER) and DNA double strand break (DSB) repair including non-homologous end joining (NHEJ) and homologous recombinational repair (HRR). Cancer cells induce changes to several key genes responsible for repair, such as ERCC1 in NER and MLH1 in MMR [4]. Several aspects of DNA repair are directly regulated by the Hedgehog (Hh) signaling pathway. This article embodies a review of the findings on the impact of Hh signaling on the DNA repair pathways in human cancer.

## 2. The Hedgehog Signaling Pathway

The Hh signaling pathway is one of the important signaling pathways that play key roles in the processes of embryonic development, carcinogenesis, maintenance of cancer stem cells (CSCs), and the acquisition of epithelial-to-mesenchymal transition (EMT) leading to metastasis [5,6,7,8,9,10,11]. The Hh signaling pathway is highly conserved from flies to humans and is essential for the normal development of an embryo [12,13]. It is initiated by binding of one of the three Hh ligands (Sonic, Indian, or Desert Hh) to the 12-pass transmembrane receptor Patched (PTCH). In the absence of ligand, PTCH represses Smoothened (SMO). In presence of ligand, this inhibition is relieved, enabling SMO to modulate a cytoplasmic complex containing Suppressor of Fused (SUFU) that modifies the three glioma-associated (GLI) transcriptional regulators through phosphorylation, sumoylation, and selective proteolysis [14]. GLI1 induces Hh target genes; GLI2 can act as an inducer or repressor depending on post-transcriptional and post-translational processing events in a cell contextual manner; GLI3 mainly serves as a repressor [15]. Vertebrate Hh signaling is further regulated by the translocation of signaling components through the cytoplasm, plasma membrane, nucleus, and the primary cilium [16]. PTCH1 is initially located in the primary cilium and SMO is within cytoplasmic vesicles. Following classical Hh ligand binding to PTCH1, SMO moves to the primary cilium, where it interacts with the GLI processing complex, eventually resulting in the nuclear translocation of the GLI transcription factors [17,18,19]. Transcription mediated by the GLI proteins is typically referred to as the canonical modulation of Hh signaling. Notably, the activation of GLI transcription (canonical) can also be initiated by other secreted proteins including osteopontin, transforming growth factor-β (TGF-β), chemokines *etc.* [20]. This type of activation of GLI by stimuli other than Hh ligands is referred to as non-classical signaling [21].

**Figure 1:**
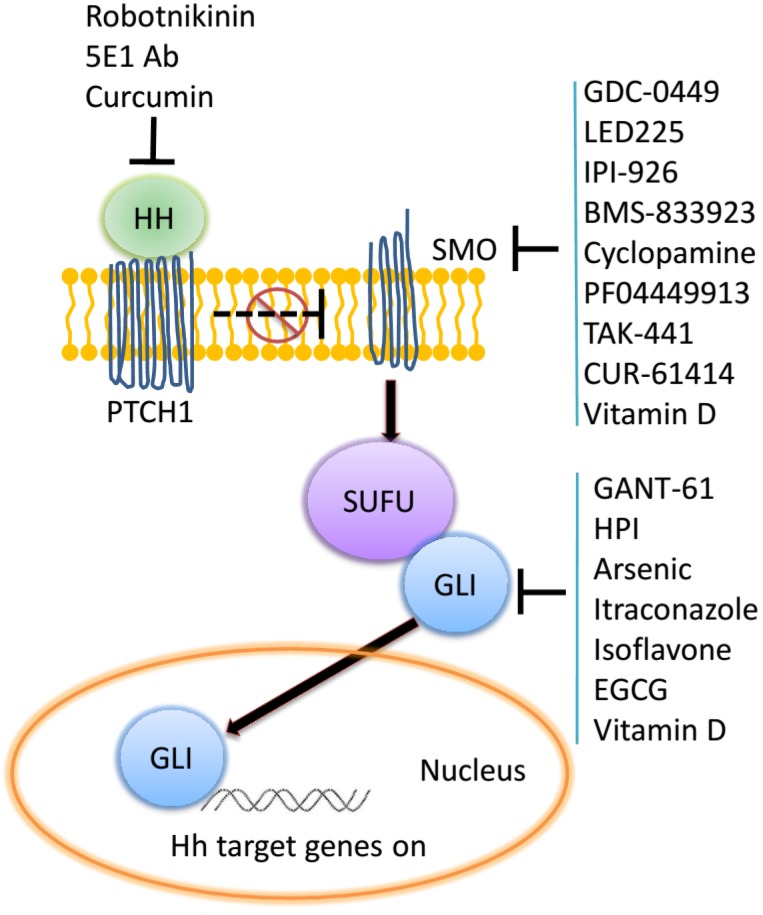
Inhibitors have been developed for their ability to block Hh signaling at various signaling nodes in the pathway. These encompass natural compounds, their chemical derivatives, a monoclonal antibody, and chemicals identified from screening libraries.

Studies of the Hh pathway, rather its functioning, have been facilitated by the availability of small molecule inhibitors that target distinct aspects of Hh signaling. The most commonly used has been cyclopamine which inhibits the activity of SMO, thereby impeding the transduction of the activation signal intracellularly and arresting the nuclear translocation of the GLI transcription factors. The pharmacologically developed SMO inhibitors are in multiple clinical trials against different cancer types. The GANT (GLI antagonists) molecules have been used thus far as research reagents to inhibit the transcriptional activity of the GLI proteins. Inhibitors of the Hh ligands include robotnikinin and the 5E1 antibody that are widely used in research to interfere in classical ligand-mediated Hh signaling (Figure 1). As one would anticipate, these inhibitors have non-overlapping effects. While the SMO inhibitors block classical (Hh ligand-dependent) signaling, they are unable to block non-classical (Hh ligand-independent) signaling that can result in activation of GLI. This is due to the fact that signaling activated by TGF-β, osteopontin and EGF is independent of the involvement of SMO. GLI inhibition by GANTs blocks all GLI-mediated transcription, thereby halting the final executionary step of this pathway [20,21,22]. Recent advances have explored other means of inhibiting signaling *via* this pathway. These include the Hh acyl transferase inhibitors that block Shh palmitoylation [23], natural GLI antagonists such as itraconazole, physalins, arsenic, vitamin D3, curcumin, and zerumbone [24]. Emerging literatures have revealed that Hh signaling affects almost all DNA repair types in human cancer. In this article we have reviewed the various types of DNA repair and the context in which they operate followed by a summary of research that has elucidated a role for Hh/GLI signaling in their regulation and the ensuing consequences. What emerges, with a focus on human cancer, is the profound impact of Hh/GLI signaling on multiple modalities of DNA repair in various cancer types. 

### 2.1. Mismatch Repair (MMR) 

As the main pathway for the repair of base-base mismatches and insertion and/or deletion loops that are formed during DNA replication [25], MMR is now recognized to be involved in the processing of DNA damage induced by several classes of clinically active (or experimental) chemotherapeutic drugs including the following: monofunctional alkylators such as temozolomide and dacarbazine); bifunctional alkylators such as the platinum analogs, cisplatin and carboplatin; and antimetabolites such as thiopurines (6-thioguanine (6-TG) and 6-mercaptopurine), and the fluoropyrimidines (5-fluorouracil and fluorodeoxyuridine) [26]. The human MMR pathway has two major components: MutS and MutL. MutS has two basic forms: MutSα and MutSβ. The MutSα heterodimer (encompassing MSH2 in a complex with MSH6) is involved primarily in the repair of base substitutions and small mismatched loops. MutSβ (encompassing MSH2 and MSH3) repairs both small and large loop mismatches. The MutL heterodimer is also present in a number of forms, including the MutLα complex (MLH1 and PMS2 proteins), the MutLβ heterodimer (MLH1 and PMS1 proteins), and MutLγ (MLH1 and MLH3 proteins). MutLα plays a primary role in the activity of MutL for mismatch correction, with MutLβ providing a minor role; MutLγ has a rather unclear role in MMR [27,28,29,30].

The MMR system is essential for the fidelity of DNA replication, whose impairment predisposes to the development and progression of many types of cancers. Findings in the recent past have provided compelling evidence for the role of Hh signaling on MMR. Inhibition of GLI1 impedes the MMR pathway. Following cDNA microarray gene profiling in human colon carcinoma cell line HT29, Mazumdar *et al.* reported that inhibition of GLI1/GLI2 by a small molecule inhibitor GANT61 down-regulated expressions of two genes (MSH6 and EXO1) involved in MMR [31,32]. While the downregulation of MMR genes by the GLI proteins may come across as paradoxical at first blush, this enables the accumulation of additional mutations in the cancer cells. Inaguma *et al.* discovered that GLI1 and GLI2 depress MMR activity in pancreatic ductal adenocarcinoma (PDAC) cells. Further investigations demonstrated that this repression is through the activation of a basic helix-loop-helix type suppressor BHLHE41 that suppresses the MLH1 promoter activity. In agreement with this finding, using immunohistochemical staining, the expression of GLI1 correlated with BHLHE41 and negatively related with MLH1 in PDAC cells and precancerous pancreatic lesions [33]. The functional implications of this relationship between MMR and Hh/GLI signaling may culminate in the regulation of the development and progression of PDAC. 

### 2.2. Nucleotide Excision Repair (NER)

NER is capable is repairing a spectrum of DNA lesions. The types of DNA damage repaired by NER include bulky DNA adducts (caused by chemicals such as aromatic amines including acetyl-aminofluorene, and nitrosamines such as MNNG) and [34] crosslinking agents (e.g., cisplatin). NER also effectively repairs UV-induced lesions, oxidatively damaged DNA, [35] and can recognize reactive products of lipid peroxidation [36]. There are four steps in the repair initiated by NER: recognition/pre-incision, incision, gap-filling, and structural repair [37,38,39,40]. First, the protein complexconsisting of XPA and replication protein A (RPA) recognize the damage. Then, the XPA-RPA proteins interact with the complex (composed of excision repair cross-complementing 3 and 2 (ERCC3 and ERCC2) or XPB and XPD and other gene products), which bears helicase activity. ERCC1-XPF and XPG, comprise the remaining subunits of the exonuclease or major enzyme complex [41]. Incision requires an exonuclease-dependent, ATP-dependent opening of the damaged double stranded DNA [37,38,39,40]. The location of the incision is adduct-dependent. The final two steps (gap-filling and structural repair) are completed by polymerase d and e, proliferating cell nuclear antigen (PCNA), replication factor C (RFC), DNA ligase, and other associated proteins [37,38,39,40]. A couple of distinct studies have brought to light the determinative role of Hh signaling in NER. The two studies used different approaches to impede Hh signaling. Inhibition of GLI1/GLI2 by GANT61 or expression of the repressor GLI3 down-regulated the expression of NER-related genes in the human colon carcinoma HT29 cell line (31). These genes included DNA ligase I (LIG1) and KIAA0101 (encodes PCNA-associated factor which binds PCNA) [42]. Another pioneering study by Kudo *et al.* found that knocking down GLI1 inhibited the up-regulation of ERCC1 and XPD, both of which are essential to NER in cisplatin-resistant human ovarian cancer cells [43]. In both these studies dampening the effects of GLI activity had profound beneficial effects on restoring sensitivity to chemotherapeutic agents in cells that were otherwise drug resistant. 

### 2.3. Direct Repair

In the direct repair family of mammalian cells, O6-methylguanine-DNA methyltransferase (MGMT) protein is the main component which removes alkyl groups from the O6 position of guanine and to a lesser extent from the O4 position from thymine [44,45,46,47]. Thus far, while there is no conclusive, functional report about the impact of Hh signaling on direct repair, Cui *et al.* have reported that MGMT was one of the multidrug resistance genes that was downregulated upon blocking Hh signaling in glioma [48]. Evidence suggests that MGMT may be transcriptionally regulated by Gli1 by virtue of GLI-binding sites in its regulatory region [49,50]. Whether impeding Hh/Gli activity restricts MGMT expression is not yet known. Thus, the translational effects of Hh/GLI signaling on direct repair remain to be demonstrated.

### 2.4. Base Excision Repair (BER)

The BER system repairs DNA lesions and strand breaks generated by mutagens [51]. BER removes nonbulky base damage induced by endogenous and exogenous adducts. Endogenous base damage entails oxidative base modifications from reactive nitrogen and oxygen species generated in cells during normal cellular respiration. Cells also accumulate reactive oxygen and nitrogen as a result of oxidative stress from ischemia or chronic inflammation [52]. The repair of nonbulky damaged bases, abasic sites, and DNA single-strand breaks (SSBs) induced by ionizing radiation, alkylating drugs, and antimetabolites (thiopurines, fluoropyrimidines, and halogenated thymidine analogus) is also predominantly mediated by BER [53,54]. During the activity of BER the damaged base is removed by a glycosylase, the phosphodiester backbone is cleaved by an apyrimidinic/ apurinic (AP) endonuclease or glycosylase/lyase and a complementary nucleotide is inserted by a polymerase. Finally, ligation of the DNA backbone restores the native structure and sequence [40]. Abrogating the transcriptional activity of GLI with GANT61 in human colon carcinoma HT29 cells down-regulated expression of genes related to BER [31]. These genes included 5'Flap endonuclease (FEN1), uracil DNA glycosylase (UNG), DNA ligase I (LIG1) and KIAA0101. In human ovarian cancer cells knocking down GLI1 inhibited the up-regulation of the BER essential XRCC1 gene [43]. This block in XRCC1 was concurrent with a loss of upregulation of c-jun with a simultaneous change in the phosphorylation pattern of the c-jun protein, with a shift from a Ser63/73 dominant pattern, to a Thr91/93 dominant pattern. Collectively the findings implicate a notable effect of Hh signaling on activation of BER.

### 2.5. DNA Double Strand Break Repair (DSB)

DSBs are typically induced by intrinsic sources like the byproducts of cellular metabolism or by extrinsic sources like X-rays, γ-rays and chemotherapeutic drugs. Mammalian cells employ two mechanisms for DSB repair: NHEJ and HRR. 

***NHEJ*** can function in all phases of the cell cycle and is the predominant repair pathway in mammalian cells. During NHEJ, the two broken ends of DNA are simply pieced together, sometimes after limited processing of the DNA ends, resulting in quick, but error-prone, repair [55]*.* NHEJ at the very least, requires the DNA-end binding Ku complex (initially recognizes the DNA break), a protein kinase DNA-PKcs (signals the presence of a break and activates repair proteins at the break), potential DNA-end processing enzymes (e.g., Artemis), and the XRCC4–Ligase IV complex (re-ligates the broken DNA ends). Targeting the Hh pathway at different levels in distinct cancer cell types has brought to light the important functions of Hh/GLI signaling in modulating NHEJ. While treatment with the GLI transcription activity inhibitor GANT61 down-regulated expression of the FEN1 gene related to NHEJ in colon carcinoma cells [56], Wu *et al.* found that the SMO inhibitor cyclopamine inhibited expression of radiation-induced DNA DSB repair proteins such as DNA-PKcs and Ku70 (NHEJ) in human pancreatic cancer cell lines (KRAS-wt Colo357 and KRAS-mutant SW1990). Cyclopamine treatment enhanced γH2AX foci in pancreatic cancer cells exposed to irradiation; this effect was rescued by EGF. Thus, in this system, cyclopamine enhanced the radiosensitivity of human pancreatic cancer cells, in part, through suppression of an EGFR-dependent pathway, indicating a rational approach in combination with radiotherapy [57]. There has been the identification of an alternate form of end-joining. The mechanism is intermediate between HR and NHEJ. This form of DSB is called microhomology-mediated end-joining (MMEJ) since it is dependent upon short sequences of a few homologous base pairs (microhomologies). The MMEJ repair mechanism can cause deletions of sequences from the strand being repaired. Thus, this pathway bears the capacity to inherently introduce errors [58].

***HRR*** HRR is a more accurate method of repair. In this process information is copied from an intact homologous DNA duplex; however, as HRR requires the presence of an intact sister chromatid, this method of repair can only operate in the S/G2 phases of the cell cycle in mammalian cells [59]*.* In addition to canonical HRR, there is an alternate form called single strand annealing (SSA) that repairs DSBs between two direct repeat sequences flanking the ends of the DSB. This results in deletion of sequence between the two repeats. Given that a major part of the human DNA comprises repeat sequences, the SSA pathway may play an important role in DNA repair and mutagenesis [60]. While NHEJ is the predominant repair pathway in mammalian cells, HRR appears to be the predominant mechanism of repair in yeast [61]. The repair of interstrand cross links (ICL) induced by chemotherapeutic agents such as cisplatin can invoke the HRR or NHEJ mechanisms and also the translesion repair system (TLS). ICL involves several nucleases, polymerases, and gene products of the Fanconi’s Anemia (FA) gene. As such, disruption in the FA gene can severely impact cancer predisposition [62,63].

In pancreatic cancer cells cyclopamine inhibited radiation-induced DNA DSB (HRR) repair proteins such as p-ATM [57] while GANT61 treatment of colon carcinoma cells caused down-regulation of expressions of genes (RAD51, RAD51C, RAD54B, RAD54L and the SNF2/RAD54 family member HELLS ) essential to HRR [31,64]. Using the same approach in HT29 and GC3/c human colon carcinoma cell lines, Shi *et al.* confirmed that following inhibition of Hh signaling using GANT61, several genes involved in HRR were down-regulated. These included RAD51C (XRCC3), RAD54B, RAD51 and HELLS [65]. Patients with basal cell nevus syndrome (BCNS or Gorlin syndrome) carry germline mutations in one allele of *PTCH1* that leads to unrestrained activation of GLI1 and an ensuing predisposition for spontaneous tumorigenesis. These patients are also at highly increased risk of tumor development in areas exposed to ultraviolet or ionizing radiation (IR). IR-induced DSB activates ATM and ATR-regulated DNA repair involving activation of the protein kinase Chk1 that regulates DNA repair, stabilizes stalled replication forks, and triggers the S-phase checkpoint. Leonard and colleagues used a Ptc1^+/−^ mouse model to recapitulate these phenotypes. The cerebella of irradiated Ptc1^+/−^ mice showed selective reduction in Chk1 activation, a phenotype also seen when Gli1 was overexpressed. This led to S-phase checkpoint defects and increased accumulation of IR-induced chromosome aberrations, suggesting that aberrant Hh pathway activation usurps a mechanism that is important for maintaining genomic stability [66]. Agyeman *et al.* have demonstrated that in HT29 cells inhibition of GLI1/GLI2 by GANT61 induced DNA DSBs marked by γH2AX nuclear foci and activation of MDC1 and NBS1 (involved in NHEJ and HRR, in particular, recognition and signaling of DSBs within chromatin and activity at replication forks) [67,68,69,70]. This study brought to light a sequential order of events that are elicited in cells when Hh/GLI activity is inhibited. Specifically, while early S-phase DNA damage warrants the activity of ATM, a transient intra S-phase checkpoint needs the recruitment of MDC1 to sites of DNA breaks marked by H2AX and needs the availability of NBS1. As such, reducing the levels of H2AX under conditions of GLI inhibition caused increased cell death [70]. As summarized above, most studies have demonstrated that inhibition of GLI1 interferes with DSB repair. As such inhibition of Hh/GLI signaling at the level of SMO or the receptor PTCH or GLI (using GANT61) notably engages the DSB repair pathway (both NHEJ and HRR).

## 3. Crosstalk among the DNA Repair Pathways

**Figure 2:**
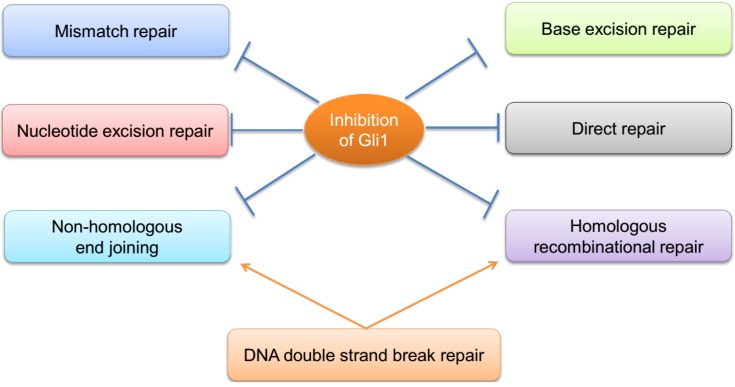
Inhibition of Hh/GLI signaling interferes with all DNA repair pathways.

A DNA lesion could initially be recognized by many repair proteins, which indicates a crosstalk among MMR, BER and NER. Interactions between different pathways are not only competitive, but also complementary and co-operative. MMR proteins facilitate very-short patch repair (a type of BER) [71,72,73] while NER, which appears to have no specific requirement for its substrate, may repair lesions left over by BER and MMR. Reversible binding of MMR and NER proteins to DNA lesions may give BER proteins, which have high affinity for specific lesions, an opportunity to interrogate and remove damaged bases. In all, inhibition of Hh/GLI1 signaling can repress almost all of the DNA repair types (MMR, NER, BER, DSB repair including NHEJ and HRR). Further studies need to be done to determine whether inhibition of GLI1/2 can functionally repress direct repair (Figure 2). 

## 4. Hedgehog Signaling and Escaping Apoptosis—A Fallback Mechanism

As irreparable DNA damage persists beyond the repair capacity of previously discussed mechanisms, cellular signals are transmitted to undergo apoptosis to prevent further permanent damage. Apoptosis is predominantly induced by the intrinsic and extrinsic pathways that ultimately result in the activation of caspase 3 leading to DNA degradation and formation of apoptotic bodies [74]. Apoptosis is regulated by the Bcl-2 family of proteins whose members both control pro- and anti-apoptotic events. Published literature portrays the influence of Hh signaling on apoptosis evasion in different cancer types. Several reports have shown that inhibition of GLI1 [75] or SMO [76,77] in pancreatic cancer through siRNA or SMO inhibitors increased apoptosis by upregulating caspase 3. In ovarian cancer, aberrant Hh activation is associated with increased levels of anti-apoptotic protein Bcl-2 [78]; inhibiting SMO induced apoptosis [79]. In glioma, knocking down Gli1 reduced Bcl-2 expression and increased apoptosis [80] and blocking SMO enhanced apoptosis [81]. In keratinocytes, the upregulation of Bcl-2 was determined to be transcriptionally regulated by Gli1, an event that was antagonized by Gli3 [82]. Thus, in addition to the ability of Hh signaling to alter the DNA repair machinery in cancer, it also allows cancer cells to escape apoptosis, the backup mechanism, in case DNA repair fails. Thus, by enabling cells to escape apoptosis, Hh signaling enriches mutations in cancer cells, selectively permitting tumor evolution, thereby making the pathway of profound importance to cancer, in particular progression and metastasis. 

## 5. Conclusion and Perspectives 

Up to now, almost all of investigations have revealed that inhibition of Hh/GLI activity reduces DNA repair activity in human cancer, suggesting that GLI1 and GLI2 may play an important role in protecting cellular DNA from lethal types of DNA damage. Exogenous DNA damage encountered by human cells is brought about by chemotherapeutic agents and/or IR. IR and chemotherapeutic DNA-damaging agents are an important component of today’s cancer chemotherapeutic regimens; however, current cancer treatment is often ineffective because of cellular resistance. Resistance to chemotherapies is governed by at least two molecular processes: DNA repair and cellular accumulation/metabolism of drug. Cellular resistance can potentially be alleviated by the use of agents that inhibit DNA repair or agents that increase cellular accumulation of active drug. Whether through ligand-dependent or ligand-independent modes, the activation of GLI-mediated transcription induces chemo-resistance in part by increasing drug efflux in an ABC transporter-dependent manner [20,21,83]. GLI1 also plays a pivotal role in cellular accumulation of cisplatin in cisplatin-resistant A2780-CP70 human ovarian cancer cells [84]. Pretreatment of cisplatin-resistant human ovarian cancer cell line A2780-CP70 with anti-GLI1 shRNA resulted in supra-additive cell killing with cisplatin, shifting the cisplatin IC50 (half maximal inhibitory concentration) from 30 mM to 5 mM [43]. In a separate study Tripathi *et al.* demonstrated that inhibition of GLI1 sensitizes human lung tumor cells A549 and HT299 to DNA Topoisomerase 1 inhibitors. DNA topoisomerase 1 (Top1)-inhibiting anticancer agents such as camptothecin (CPT) analogs (irinotecan and topotecan) show a broad spectrum of antitumor activity against colorectal, lung, and ovarian cancer either alone or in combination with other agents. The implications of this are immense in that the inhibition of GLI1 in combination with Top1-targeting agents would be an effective therapeutic modality in GLI1-expressing tumors [85,86,87,88]. All of these combined effects of Hh/GLI signaling on DNA repair, drug efflux and cellular accumulation of drug suggest an important role of this signaling pathway in chemo-resistance, bringing to the forefront this pathway as a very important target to overcome multi-drug resistance (MDR) and increase chemotherapeutic response in the treatment of cancer. Given the obvious emergence of resistance to Hh inhibitors, mainly involving resurgent mutations in SMO, there have been exciting recent advances in the identification of Glabrescione B as the first small molecule that binds Gli1 zinc finger (interfering with its DNA interacting capacity) [89] and JQ1, a BET bromodomain protein BRD4 inhibitor that inhibits transcription events by Gli1 [90]. Given the tumor cell killing achieved by the Hh inhibitors, the role of the Hh inhibitors on the re-modeling of the stromal microenvironment cannot be sidelined [76,91]. While the stromal component plays a vital role in hematopoietic malignancies, pancreatic and other solid tumors, it also protects tumor cells from succumbing completely to the inhibitors [21,92,93,94,95]. Further investigations on formulating effective combination of inhibitors of Hh/GLI signaling and conventional cytotoxic chemotherapeutic regimens or IR for the treatment of cancer are much needed. Besides Hh signaling, other signaling pathway like canonical Wnt/β*-catenin* [96] and TGF-β*1* [97,98] can also enhance the activity of DNA repair systems in cancer. Likewise, concurrent treatment with an inhibitor of these signaling pathways is a potential therapeutic strategy for increasing the response of cancer to chemotherapies and radiotherapies. 
